# Early identification of carotid vulnerable plaque in asymptomatic patients

**DOI:** 10.1186/s12872-020-01709-5

**Published:** 2020-10-01

**Authors:** Yungen Jiao, Yahong Qin, Zhengang Zhang, Hao Zhang, Haiwei Liu, Chen Li

**Affiliations:** 1 Department of Cardiology, The Affiliated Hospital of Yangzhou University, Yangzhou University, 45# Taizhou road, Yangzhou, 225000 Jiangsu Province China; 2 521 Hospital of Norinco Group, 12# Zhangba East Road, Xi’an, 710065 Shaanxi Province China

**Keywords:** Carotid artery ultrasound, Atherosclerosis, Unstable plaque, Vulnerable plaque, Serum marker, Early recognition

## Abstract

**Background:**

This study was to explore the influencing factors of atherosclerotic plaque formation and stability in patients with asymptomatic carotid atherosclerotic plaques, so as to identify the vulnerable plaques at early stage, and then find high-risk group of cardio-cerebrovascular events for early clinical intervention to reduce related mortality and disability.

**Methods:**

A total of 302 enrolled patients with asymptomatic carotid atherosclerotic plaques were divided into 3 groups based on the results of carotid artery color Doppler ultrasound: atherosclerotic unstable plaque (UP) group, atherosclerotic stable plaque (SP) group, and control group without plaques. Serum markers were measured by ELISA. *χ*^*2*^ test, *t* test, Pearson correlation analysis, and Logistic multivariate regression analysis were used in the analysis, and *P* < 0.05 was considered statistically significant.

**Results:**

It revealed that high MMP-9, LOX-1and YKL-40 were independent risk factors for unstable plaque formation. The area under the curve (AUC) of serum markers combined with MMP-9, LOX-1 and YKL-40 was 0.850, with sensitivity 87.67%, specificity 81.13%, and diagnostic accuracy 84.92%, which was significantly better than the individual diagnostic efficacy of other three factors. The accuracy rate of Crouse Plaque Score (CPS) in the diagnosis of vulnerable plaques was 61.90%, the 10-year ICVD diagnosis accuracy rate was 56.75%, and the diagnostic accuracy of serum markers was significantly better than CPS and 10-year ICVD.

**Conclusion:**

Noninvasive cervical color Doppler ultrasound combined with serum markers MMP-9, LOX-1 and YKL-40 have significant early recognition effect on asymptomatic carotid vulnerable plaque patients.

## Background

Atherosclerosis (AS) is the root cause of cardiovascular and cerebrovascular diseases, including coronary artery disease and stroke. More than 4 million people die of cardiovascular and cerebrovascular disease every year in China, ranking the first in fatal diseases [1]. Atherosclerosis is an independent risk factor for cardiovascular and cerebrovascular diseases. Plaque rupture and subsequent thrombosis are the main causes of myocardial infarction and stroke. With the continuous improvement of people’s living standards, the occurrence of cardiovascular and cerebrovascular events is showing a younger trend, and some senile diseases such as hypertension, cerebral infarction, and myocardial infarction appear earlier, increasing the mortality and disability rate of Chinese people. Therefore, early detection and effective intervention of patients with atherosclerotic cardiovascular and cerebrovascular disease (ASCCVD) is an important measure to reduce cardiovascular and cerebrovascular disease and its complications.

ASCCVD includes stroke, coronary heart disease (CHD), and peripheral arterial disease (PAD) [2]. The basic pathological change of ASCCVD is atherosclerosis. AS is a systemic, long-term, slowly progressing inflammatory disease. The basic lesions are lipid deposition in the intima of the artery, focal fibrosis of the intima, and atheromatous plaque formation. Atherosclerotic plaques are divided into stable plaques and unstable plaques, unstable plaques are also called vulnerable plaques. The methods of identifying vulnerable plaques early include [3, 4]:multi-slice spiral CT, nuclear magnetic resonance, radionuclide imaging, optical coherence tomography, etc. Digital subtraction angiography (DSA) is the “gold standard” for the diagnosis of arterial disease, but its application is limited due to its invasiveness and high cost. Carotid artery color Doppler ultrasound is the preferred imaging method for clinical examination of atherosclerosis currently, it has the characteristics of low price, non-invasive, easy to operate, radiationless, and repeatable, which makes it widely used in clinical applications and almost no contraindications in the tested population. The superficial position, large diameter and relatively fixed position of carotid artery make it particularly suitable for non-invasive imaging. At the same time, the carotid artery is an early involved blood vessel in atherosclerotic lesions. Carotid atherosclerosis is not only related to the severity ofischemic heart and brain vascular disease, and it is also a window into the atherosclerotic state of other blood vessels throughout the body. Therefore, early detection of the condition of the inner wall of the carotid artery by B-ultrasound has important clinical significance and can well predict the patient’s risk of ASCCVD [5, 6]. The serum markers of vulnerable plaques in circulation are sensitive, highly operable, accurate, acceptable to patients and guide clinical decisions for clinicians, and can predict the risk of acute cardiovascular and cerebrovascular events and complications in patients.

There are few studies on early identification and intervention of carotid vulnerable plaques in patients with asymptomatic health checkups and there is no standardized diagnosis and treatment recommendations at present. Patients only undergo clinical intervention after they develop cerebral infarction or myocardial infarction. That is an important reason for the high mortality and disability of ASCCVD patients. The purpose of this study is to explore the influencing factors of atherosclerotic plaque formation and stability in patients with asymptomatic carotid atherosclerotic plaques. At the same time, we will accurately identify the vulnerable plaques and find the patients of asymptomatic ASCCVD earlier through combining the non-invasive carotid artery ultrasound and the detection of serum plaque stability markers so as to conduct the clinical intervention, delay the progress of plaque, avoid thrombosis and prevent the occurrence and development of ischemic cardiovascular and cerebrovascular diseases.

## Methods

### Study subjects

From March 2019 to September 2019, patients with atherosclerotic plaques found by two-dimensional ultrasound in the physical examination center of Affiliated Hospital of Yangzhou University were selected as the study objects. According to the plaque nature, they were divided into UP group of 146 cases and SP group of 106 cases. Another 50 patients without plaques were selected as the control group. Inclusion criteria: 1. Conventional two-dimensional ultrasound showed carotid plaque; 2. Patients agreed and voluntarily accepted the investigation and follow-up, the clinical data was complete and valuable, signed the consent. Exclusion criteria: 1. patients with cerebral infarction, coronary heart disease and peripheral artery disease with definite diagnosis; 2. patients with malignant tumor, liver and kidney failure and serious infection; 3. any systemic immune disease; 4. pregnant and lactating women; 5. patients taking anticoagulants orally or intravenously for nearly 1 month.

### Patients data collection

The demographic data such asmedical history, personal history and family history were collected. Smoking is defined as: smoking at least one cigarette per day, and smoking for more than 1 year continuously. Those who have a clear history of smoking are classified as smokers, and those who have never smoked are classified as non-smokers [7]; Drinking is defined as: drinking at least 50 g per week, and drinking for more than 1 year continuously. Those who have a clear drinking history are all classified as drinking population, and those who drink occasionally are non-drinking population; Body mass index (BMI, kg/m^2^): < 24 is normal, 24–28 is overweight, and ≥ 28 is obesity; The diagnosis standard of hypertension is systolic blood pressure ≥ 140 mmHg and/or diastolic blood pressure ≥ 90 mmHg or there is a clear history of hypertension and currently taking antihypertensive drugs; The diagnosis standard of T_2_DM is fasting blood glucose > 7.0 mmol/L or taking hypoglycemic drugs or insulin hypoglycemic treatment. Take a test for fasting blood for ≥8 h of venous blood to detect fasting blood glucose (FPG), total cholesterol (TC), triglycerides (TG), HDL-C, LDL-C, blood uric acid (UA) and creatinine (Cr) to collect the basic clinical biochemical data, while calculating carotid plaque score (CPS) [8] and 10-year risk of ICVD [7, 9].

### Carotid artery ultrasound

All subjects were rested for 10 min before getting the carotid ultrasound, and change to the supine position so as to fully expose their lateral neck, meanwhile, tilting their head to the contralateral side, and the procedure is operated by a specialist of ultrasound doctor, using Chinese Sonoscape S20 color Doppler ultrasound system. The standard common high-frequency probe is used, and the probe rate is 5 ~ 10 MHz. Both common carotid arteries and their bifurcations, and internal carotid artery are detected in turn, and the intimal thickness of the tube wall is observed. For normal blood vessels, intimal thickening is 1.0 mm < IMT ≤ 1.2 mm, and IMT > 1.2 mm is defined as plaque. Carotid atherosclerotic plaques are classified according to plaque location, size, and echo characteristics: ① stable plaque (high or strong echo plaque); ② unstable plaque (low echo, isoechoic, mixed echo plaque).

### Serum marker detection

Take 5 ml of peripheral venous blood from the subject in the morning, add it to a anticoagulation tube, centrifuge at 3000 r/min for 10 min, separate the supernatant, store in a − 80 °C refrigerator, and measure serum MMP-9, LOX-1, YKL-40, PAPP-A (MMP-9, LOX-1, YKL-40, PAPP-A human ELISA kits, purchased from Shanghai Enzyme Biotechnology Co, Ltd) by quantitative enzyme-linked immunosorbent (ELISA) method. Serum MMP-9, LOX-1, YKL-40, and PAPP-A contents were determined according to the specified steps of the ELISA kit.

### Statistical analysis

SPSS 24.0 statistical software was used for data analysis. The measurement data in accordance with normal distribution were expressed as mean ± standard deviation (x ± s). All continuous variable data were compared by ANOVA or Student’s t-test. The count data were expressed by percentage (%) and chi-square test was performed. For non-normally distributed variables, we used the median and inter-quartile range to represent. Influencing factors of plaque formation and plaque stability wereevaluated using univariate and multivariate regression analyses. ROC curve was used to analyze the independent and combined diagnostic efficacy of serum markers. *P* values were two-sided, and *P* values ≤0.05 were considered as statistically significant.

## Results

### Univariate analysis

Single factor analysis shows that age, gender, smoking, hypertension, T_2_DM, HDL-C, MMP-9, LOX-1, YKL-40, PAPP-A, Lipids ratio and 10-year ICVD values have statistically significant difference (*P* < 0.05) between patients without plaque and those with plaque, as shown in Table 1. Smoking, T_2_DM, HDL-C, LDL-C, CPS, MMP-9, LOX-1, YKL-40, PAPP-A, and 10-year ICVD are significantly higher in the unstable plaque group than the stable plaque group, HDL-C is lower in the unstable plaque group than the stable plaque group (*P* < 0.05), see Table 1.

**Table 1:** Single factor analysis of various indicators for medical examiners

Group	Unit	Plaque Group (252)	No Plaque group (50)	*T* or *χ*^*2*^*/P*	Unstable plaque group (146)	Stable plaque group (106)	*T* or *χ*^*2*^*/P*
Age	years	62.43 ± 11.23	57.64 ± 10.53	2.784/0.006	62.80 ± 11.61	61.92 ± 10.72	0.611/0.542
BMI	kg/m2	24.70 ± 2.65	24.35 ± 3.05	0.814/0.416	24.75 ± 2.59	24.61 ± 2.75	0.409/0.683
TG	mmol/L	2.58 ± 2.13	2.25 ± 0.10	1.692/0.093	2.65 ± 2.14	2.48 ± 2.12	0.613/0.540
TC (CHO)	mmol/L	4.81 ± 0.10	4.88 ± 0.79	−0.469/0.639	4.87 ± 0.93	4.73 ± 1.07	1.131/0.259
HDL-C	mmol/L	1.23 ± 0.29	1.33 ± 0.32	−2.348/0.020	1.19 ± 0.27	1.27 ± 0.32	−2.135/0.034
LDL-C	mmol/L	2.67 ± 0.81	2.66 ± 0.65	0.141/0.888	2.77 ± 0.84	2.54 ± 0.75	2.233/0.026
UA	mmol/L	360.56 ± 80.53	353.18 ± 84.29	0.587/0.558	357.26 ± 74.89	365.10 ± 87.87	−0.744/0.458
Cr	mmol/L	76.62 ± 16.76	78.02 ± 19.01	−0.528/0.598	76.55 ± 17.88	76.72 ± 15.17	−0.079/0.937
MMP-9	ng/ml	292.25 ± 106.11	243.48 ± 116.30	2.921/0.004	333.84 ± 92.42	234.97 ± 96.96	8.212/0.000
LOX-1	pg/ml	212.82 ± 59.23	189.15 ± 53.00	2.625/0.009	223.44 ± 59.83	199.15 ± 54.58	3.301/0.001
YKL-40	ng/ml	226.17 ± 68.10	192.79 ± 57.10	3.246/0.001	237.08 ± 67.24	211.15 ± 66.70	3.031/0.003
PAPP-A	ng/ml	5.86 ± 3.22	2.86 ± 1.34	10.804/0.000	6.45 ± 3.22	5.06 ± 3.06	3.458/0.001
10-year ICVD	%	10.90 ± 10.29	5.24 ± 5.51	5.585/0.000	12.43 ± 11.58	8.79 ± 7.74	2.989/0.003
WC	cm	84.08 ± 7.96	83.06 ± 9.14	0.814/0.416	84.26 ± 7.77	83.84 ± 8.24	0.409/0.683
Lipids ratio	–	2.26 ± 0.79	2.07 ± 0.59	1.652/0.100	2.41 ± 0.86	2.06 ± 0.63	3.704/0.000
Gender	Male/Famale	220/32	38/12	4.281/0.039	124/22	96/10	1.759/0.185
Smok	Yes/No	120/132	13/37	7.912/0.005	81/65	39/67	8.598/0.003
Drink	Yes/No	38/214	10/40	0.756/0.385	23/123	15/91	0.480/0.489
Hypertension	Yes/No	176/76	21/29	14.259/0.000	106/40	70/36	1.257/0.262
T_2_DM	Yes/No	90/162	7/43	9.023/0.003	61/85	29/77	5.564/0.018
CPS	mm	–	–	–	3.92 ± 2.31	2.82 ± 1.43	4.672/0.000
Exercise	Yes/No	143/109	27/23	0.128/0.721	80/66	63/43	0.539/0.463
AF	Yes/No	4/248	0/50	0.804/0.370	3/143	1/105	0.486/0.486

### Logistic multivariate regression analysis

Taking the presence or absence of unstable plaque as the dependent variable, the positive result indicators in Table 1 are the independent variables, and the results of logistic multivariate regression analysis shows that T_2_DM, high MMP-9, LOX-1, YKL-40 CPS, andLipids ratioare independent risk factors for unstable plaque formation (T_2_DM: OR = 0.399, *P* = 0.009; MMP-9: OR = 1.015, *P* = 0.000; LOX-1: OR = 1.008, *P* = 0.008;YKL-40: OR = 1.007, *P* = 0.013; CPS:OR = 1.322, *P* = 0.006; Lipids ratio: OR = 2.312, *P* = 0.001), which is shown in Table 2.

**Table 2:** Multivariate Logistic Regression Analysis of Physical Examination Indicators

	Factor	*β*	*SE*	*Waldχ*^*2*^	*P*	*OR*	*95%CI*
Plaque or not	Gender	−1.082	0.518	4.359	0.037	0.339	0.123–0.936
	HBP	−1.283	0.403	10.135	0.001	0.277	0.126–0.611
	T_2_DM	−1.139	0.515	4.894	0.027	0.320	0.117–0.878
	PAPP-A	0.985	0.167	34.826	0.000	2.679	1.931–3.716
	YKL-40	0.009	0.004	5.412	0.020	1.009	1.001–1.016
Plaque stability	T_2_DM	−0.918	0.351	6.857	0.009	0.399	0.201–0.794
	MMP-9	0.014	0.002	37.256	0.000	1.015	1.010–1.019
	LOX-1	0.008	0.003	6.970	0.008	1.008	1.002–1.015
	YKL-40	0.007	0.003	6.234	0.013	1.007	1.001–1.012
	CPS	0.279	0.102	7.485	0.006	1.322	1.082–1.615
	Lipids ratio	0.838	0.258	10.54	0.001	2.312	1.394–3.834

### Analysis of ROC curves of serum markers MMP-9, LOX-1 and YKL-40

**Fig. 1:**
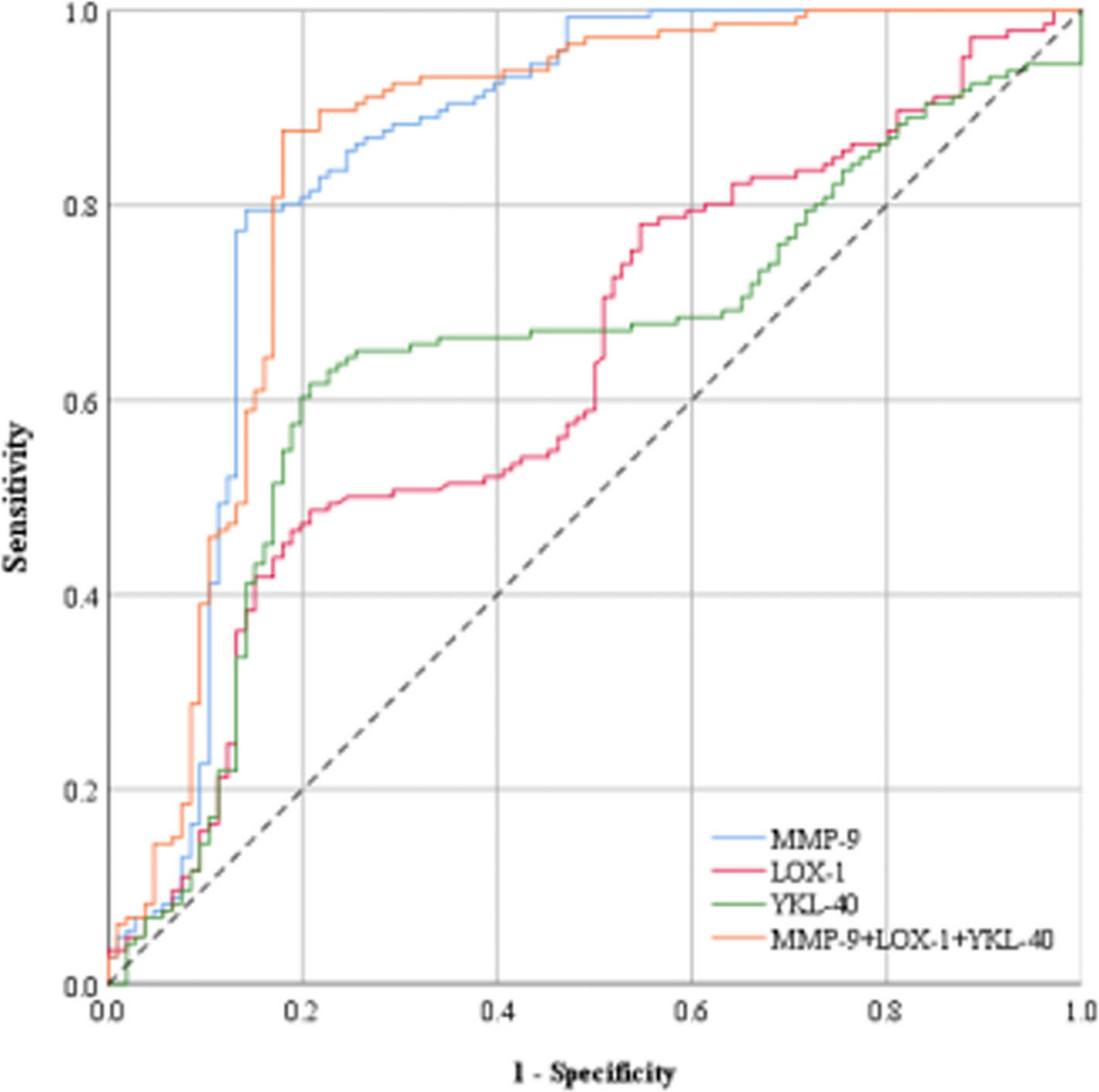
ROC curve of three factors for MMP-9, LOX-1, YKL-40 independent andcombineddiagnosis of vulnerable plaques. The ordinate is the sensitivity, representing the true positive rate, and the abscissa is the specific generation, which represents the false positive rate. The larger the area under the curve, the higher the accuracy of the diagnosis of vulnerable plaque. The curve area under the red line (0.630) represents the accuracy of LOX-1 in diagnosing vulnerable plaque. The curve area under the green line (0.646) represents the accuracy of YKL-40 in diagnosing vulnerable plaque. The area of the curve under the blue line (0.847) represents the accuracy of MMP-9 in diagnosing vulnerable plaque. The area of the curve under the orange line (0.850) represents the accuracy of the combined diagnosis of vulnerable plaques by the three markers LOX-1, YKL-40 and MMP-9

In this study, ROC curve is used for the first time to analyze the independent and combined prediction of vulnerable plaque by serum markers, and compared with the previous evaluation criteria. The binary logistic regression analysis shows that T_2_DM, MMP-9, LOX-1, YKL-40, Lipids ratio, and CPS are factors that affect plaque stability. We tested the concentrations of MMP-9, LOX-1, and YKL-40 in the serum and included them in multivariate logistic regression analysis. The ROC curve of the three factors independent and combined diagnosis of vulnerable plaque is shown in Fig. 1. The effectiveness of the three factors independent and combined diagnosis of vulnerable plaques is shown in Table 3. It can be seen that the AUC of the three factors combined to diagnose vulnerable plaques is 0.850 (*95% CI*: 0.795–0.905), the sensitivity is 87.67%, the specificity is 81.13%, and the diagnostic accuracy is 84.92%, which is significantly better than the three factors alone. Among many influencing factors, MMP-9 has the greatest impact. The accuracy of MMP-9 alone diagnosis is 80.95%, which has a good diagnosis effect. According to the results of color Doppler ultrasound of cervical blood vessels and the serum indicators of examinees, an intervention is basically confirmed necessary when MMP-9 is greater than 282.74; when MMP-9 is less than 282.74, LOX-1 is greater than 223.78, or YKL-40 is greater than 215.98, follow up observation and recheck on time are needed; when MMP-9, LOX-1, YKL-40 are all less than the critical value, it is basically determined that no intervention is necessary.

**Table 3:** Comparative analysis of the effectiveness by independent and combined diagnosis of three factors for vulnerable plaques

Variable	ROC Area	*95%CI*	Sensitivity (%)	Specificity (%)	Positive predictive value (%)	Negative predictive value (%)	Diagnostic accuracy (%)	*P*	Critical value	Positive and negative likelihood ratios	Youden index
MMP-9	0.847	0.791 ~ 0.903	88.36	70.75	80.63	81.52	80.95	0.000	282.74	18.36	0.591
LOX-1	0.630	0.560 ~ 0.700	85.62	24.53	60.98	55.32	59.92	0.000	223.78	1.94	0.102
YKL-40	0.646	0.576 ~ 0.716	81.51	25.47	60.10	50.00	57.94	0.000	215.98	1.51	0.070
MMP-9 + LOX-1 + YKL-40	0.850	0.795 ~ 0.905	87.67	81.13	86.49	82.69	84.92	0.000	–	30.57	0.688
10-year ICVD	0.590	0.520 ~ 0.661	85.62	16.98	58.69	46.15	56.75	0.014	–	1.22	0.026
Carotid plaque core	0.654	0.586 ~ 0.721	75.34	43.4	64.71	56.10	61.90	0.000	–	2.34	0.187

### ROC curve analysis of CPS, 10-year ICVD and serum markers in the diagnosis of vulnerable plaques

**Fig. 2:**
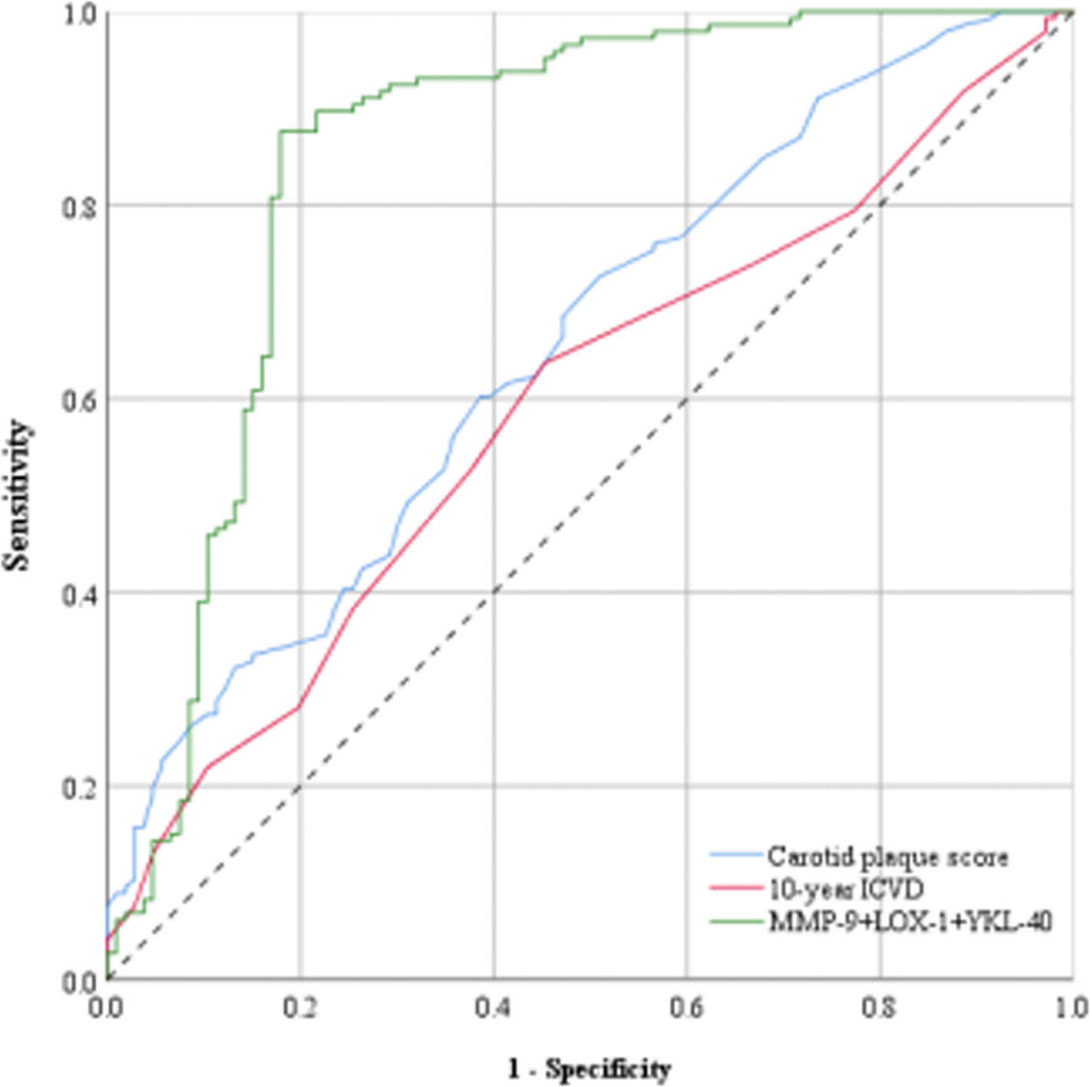
ROC curve of three kinds of vulnerable plaque diagnosis method. The ordinate is the sensitivity, representing the true positive rate, and the abscissa is the specific generation, which represents the false positive rate. The larger the area under the curve, the higher the accuracy of the diagnosis of vulnerable plaque. The area of the curve under the red line (0.590) represents the accuracy of 10-year ICVD diagnosis of vulnerable plaque. The curve area under the blue (0.654) represents the CPS diagnosis of vulnerable vulnerable plaque. The area of the curve under the green line (0.850) represents the combined diagnosis of the three serum markers LOX-1, YKL-40 and MMP-9

We compared the accuracy of three methods for predicting vulnerable plaques including serum markers, 10-year ICVD, and CPS. As shown in Fig. 2, the diagnostic accuracy of CPS was 61.90% (*95%CI*: 0.586–0.721, *P* = 0.000), the diagnostic accuracy of 10-year ICVD was 56.75% (*95%CI*: 0.52–0.661, *P* = 0.014), and the diagnostic accuracy of the serum markers studied in this article was 84.92% (*95%CI*: 0.795–0.905, *P* = 0.000). It can be seen that the combined use of serum markers MMP-9, LOX-1 and YKL-40 to predict plaque vulnerability has a better diagnostic effect on potential patients with ASCCVD.

### Early clinical intervention guided by 10-year ICVD risk assessment and serum markers

A study [10] had divided the 10-year ICVD risk assessment to guide early clinical intervention into four situations. No intervention if the absolute risk population < 5%, considering intervention when it is 5–7.5%, intervening when it is 7.5–20% and > 20%, as a way to determine the objects who need early intervention. In order to further accurately identify the high-risk population of cardiovascular and cerebrovascular events that need intervention in the early stage, we take vulnerable plaque group as the research object to explore the accuracy rate of combining serum markers and 10-year ICVD to predict high-risk ASCCVD patients. See Table 4 for more details.

**Table 4:** Early clinical intervention guided by 10-year ICVD risk assessment and serum markers

10-year ICVD risk classification	< 5%	5–7.5%	7.5–20%	> 20%
10-year ICVD assess risks separately	non intervention	consider intervention	intervention	intervention
10-year ICVD in combination with MMP-9, LOX-1, YKL-40 any>critical value	23.3% non intervention	20.5% intervention	29.5% intervention	21.9% intervention
10-year ICVD in combination with MMP-9, LOX-1 and YKL-40 were all lower than the critical value	2.7% non intervention	0.7% consider intervention	1.4% consider intervention	0.0% intervention
Differences between serum markers and 10-year ICVD	×	√	√	×

## Discussion

Vulnerable plaques are clinically defined as [11, 12] atherosclerotic plaques that are susceptible to rupture, thrombus formation and rapid progression, and are likely to develop into “criminal plaques”, ormore likely to cause “clinical events” atherosclerotic plaquesto occur. The morphological changes of vulnerable plaques mainly include: rupture of the plaque surface, components calcification and changes, thinning of the fibrous cap, formation of huge lipid cores, formation of neovascularization, plaque hemorrhage and secondary thrombosis. The changes of cellular molecular level of vulnerable plaques mainly include smooth muscle cell proliferation, macrophage aggregation, extracellular lipid aggregation, T lymphocyte aggregation, etc. Vulnerable plaque is the basis for the occurrence of angina pectoris, myocardial infarction and stroke, but atherosclerosis is usually clinically silent before the acute events caused by narrowing of arterial lumen or plaque rupture. Up to 60% of acute myocardial infarction (AMI), sudden cardiac death and stroke are the first manifestations of the disease [13]. Therefore, it is of great significance for early accurate assessment and effective intervention of vulnerable plaques [14].

In addition to the previously studied risk factors for unstable plaque formation such as hypertension, T_2_DM, and lipids, this study find that serum markers MMP-9, LOX-1, and YKL-40 are also independent risks of unstable plaque formation. CPS is an independent predictor of vulnerable plaque formation. Atherosclerotic plaques are composed of a lipid core covered with a fibrous cap, which is mainly composed of an extracellular matrix containing a large amount of collagen fibers, smooth muscle cells, and a small number of macrophages. The lipid core is composed of macrophages, smooth muscle cells, and ECM composition. The weakening of the fibrous cap due to the net degradation of the extracellular matrix is considered to be an important cause of plaque rupture. MMP-9, also known as gelatinase B [15, 16], is highly expressed in macrophage-rich atheromatous plaque regions, and its main function is to mediate the degradation and remodeling of ECM, as well as the degradation of all components of the vascular wall, so it plays an important role in the degradation of the fibrous cap in arterial plaque. Laura [17] found that MMP-9 can promote the transformation of plaque from stable to vulnerable state, increase plaque instability, and MMP-9 has certain value for the prediction, diagnosis and prognosis of acute coronary syndrome. The results of this study support the view that MMP-9 predicts vulnerable plaques, but the average value of the plaque-free group is greater than 0 ng/ml. Therefore, MMP-9 may be involved in other stages besides atherosclerotic plaque progression, such as tissue remodeling [18], inflammation [19], tumor invasion [20], wound healingc [21] and other physiological processes can increase its serum value. LOX-1 is a class E scavenger receptor, which accelerates the instability of arterial plaques by promoting lipid accumulation, inducing endothelial cell activation and dysfunction, and indirectly degrading the extracellular matrix [22, 23]. Multivariate regression analysis in this study showed that LOX-1 is an independent risk factor for vulnerable plaque in the carotid artery, which is basically consistent with the previous research results. Serum LOX-1 level can serve as an index to evaluate the properties of arterial plaque. YKL-40 [24] is an inflammatory glycoprotein without chitinase activity. It is mainly expressed by macrophages at the late stage of differentiation. It not only participates in angiogenesis, cell migration and tissue remodeling, but also aggravates plaque instability by affecting the synthesis of hyaluronic acid as well as the expression and activation of MMP-9. Li [25] studied the relationship between serum inflammatory markers and plaque properties in patients with h-type hypertension and carotid atherosclerosis confirmed that YKL-40 level was positively correlated with plaque properties. As plaque instability increased, serum YKL-40 is on the rise. Our study found that YKL-40 may exist as a potential biomarker of vulnerable plaque circulation, and it is of great significance for the evaluation of atherosclerotic plaque properties.

In 1986, J R. Crouse first proposed the concept of “plaque score” to quantitatively analyze the degree of arteriosclerosis, also known as “Crouse score” [8]. The Crouse Score defines IMT > 1.2 mm as plaques. Regardless of the length of a single plaque, the maximum thickness (mm) of each isolated plaque on both sides of the carotid artery measured by the cross-section of the probe is added to obtain the bilateral carotid artery. The sum of plaque thickness is the carotid plaque score (CPS). Many previous studies [5, 26, 27] confirmed that CPS is an independent predictor of ASCCVD, and unstable plaque is the basis of the onset of ASCCVD, in other words, both high CPS and unstable plaques can easily lead to the onset of cardiovascular and cerebrovascular diseases. The CPS reflects the instability of plaques from the side. The relationship between CPS and carotid plaque stability has not been reported at home and abroad. The results of this study find that the CPS has a strong correlation with plaque stability. The average CPS of the unstable plaque group is significantly higher than that of the stable plaque group. Multivariate regression analysis showed that the CPSis an independent predictor of vulnerable plaque (*OR* = 1.334; *95% CI*: 1.087–1.637; *P* = 0.006). This result validates the opinions of previous literature. CPS is obtained by carotid ultrasound, which has the characteristics of easy to operation, cheap price, and no radiation. Therefore, it can be used as a screening for high-risk ASCCVD population. However, it should be noted that the subjective factors of operators and the plaque definition criteria in clinical practice may affect. There are some errors in the measurement results.

In order to predict the incidence of cardiovascular and cerebrovascular diseases early, many standards and indicators have been developed. These tools can help identify high-risk populations, thereby raising people’s awareness, improving lifestyles, and even reducing morbidity and mortality. One of the simplest and most practical methods is the Framingham risk score (FRS). Since the release of FRS by Wilson et al. in 1998, FRS has become the basis for calculating the risk of adult treatment in the United States. This model can estimate the risk of cardiovascular disease in ordinary people for 10 years, and provides a convenient method for classification of low, middle and high risk groups of coronary heart disease [28]. The 10-year ICVD calculation in this study applied the improved Chinese “Ischemic Cardiovascular Disease (ICVD) 10-year Onset Risk Assessment Method”, and based on the sum of the scores of various risk factors, the absolute 10-year ICVD risk of the individual was obtained. The univariate analysis in this study showed that the average 10-year ICVD in the plaque group was significantly larger than that in the non-plaque group (*P* = 0.000). Compared with the stable plaque group, the 10-year ICVD was higher in the unstable plaque group, and we also found thatvulnerable plaques have a good correlation with the 10-year ICVD trend (*P* = 0.003). Multivariate analysis found that 10-year ICVD is not an independent factor for vulnerable plaques. If very few special samples are excluded (such as older but not smoking, hypertension, hypertension, T_2_DM, etc., it will also calculate a larger 10-year ICVD value), calculation found that 10-year ICVD is an independent predictor of vulnerable plaque. The combined detection method in this study made up for the shortcomings of 10 years of inaccurate prediction of ICVD for special cases.

In order to further screen high-risk ASCCVD patients early with more accuracy and to avoid the shortcomings of inaccurate prediction of special cases, we took patients in the vulnerable plaque group as the research object and explored the combination of serum markers and 10-year ICVD in predicting high-risk patients with ASCCVD, as shown in Table 4. It is recommended to combine 10-year ICVD and serum markers to guide early intervention. Ifthe 10-year ICVD risk is < 5%, early intervention is not recommended regardless of the amount of serum markers; The risk of ICVD in 10 years is 5–7.5%, and any marker of MMP-9, LOX-1 and YKL-40 is higher than the critical value, it is suggested to intervene; When MMP-9, LOX-1, YKL-40 < critical value, considering intervention; The 10-year ICVD risk is 7.5–20% and any marker of MMP-9, LOX-1 and YKL-40 ishigher than the critical value, it is recommended for intervention; MMP-9, LOX-1, YKL-40 < critical value, considering intervention; The 10-year ICVD risk> 20%, and early intervention is recommended regardless of serum markers.

### Limitation

This study has some limitations. First, the experimental sample size is limited: we will continue to supplement and improve in the future experiment process; Second, because the plaque thickness is calculated in millimeter (mm), there may be some subjective factors that may produce certain errors in the measurement, which will affect the CPS results. However, each measurement is was not completed by a single person, but participated by many people. We believe that our results are true and reliable.

### Future directions

In the future, we can increase the number of cases, carry out long-term intervention and follow-up for asymptomatic patients with cardiovascular and cerebrovascular diseases identified early. We can also further carry out cervical vascular contrast-enhanced ultrasound to conduct qualitative and quantitative analysis of plaque, explore more serological markers related to vulnerable plaque, and provide more reference for the prediction of cardiovascular and cerebrovascular diseases combined with existing relevant indicators, so as to truly achieve “early onset” Early diagnosis, early treatment “.

## Conclusions

We find that MMP-9, LOX-1 and YKL-40 are independent risk factors of unstable plaque formation, which have significant early recognition effect on asymptomatic carotid vulnerable plaque patients. Our study also find that the combination of the above three markers in the diagnosis of vulnerable plaque is significantly better than the independent predictive value of single marker, which can provide a new idea for early and accurate identification of high-risk groups of cardiovascular and cerebrovascular events.

## Data Availability

The datasets used and/or analysed during the current study are available from the corresponding author on reasonable request.

## Abbreviations

UP: Unstable plaque
SP: Stable plaque
MMP-9: Matrix metalloproteinases-9
LOX-1: Lectin-like oxidized low-denisty lipoprotein receptor-1
YKL-40: Human cartilage glycoprotein-39
PAPP-A: Pregnancy-associated plasma protein-a
CPS: Carotid artery plaque score
10-ICVD: Ischemic cardiovascular diseaseof 10 year
AS: Atherosclerosis
ASCCVD: Arteriosclerotic cardiovascular andcerebrovascular disease
ECM: Extracellular matrix
FRS: Framingham risk score
WC: Waist circumference
AF: Atrial fibrillation
Lipids ratio: LDL-c/HDL-c
